# Pathogenic Hydrogel? A Novel-Entrapment Neuropathy Model Induced by Ultrasound-Guided Perineural Injections

**DOI:** 10.3390/ijms22073494

**Published:** 2021-03-28

**Authors:** Ming-Yen Hsiao, Ya-Wen Wu, Wen-Shiang Chen, Yu-Ling Lin, Po-Ling Kuo, Chueh-Hung Wu

**Affiliations:** 1 Department of Physical Medicine and Rehabilitation, College of Medicine, National Taiwan University, Taipei 10048, Taiwan; myhsiao@ntu.edu.tw (M.-Y.H.); wenshiang@gmail.com (W.-S.C.); cj801107@gmail.com (Y.-L.L.); 2 Department of Physical Medicine and Rehabilitation, National Taiwan University Hospital, Taipei 10048, Taiwan; 0607091@gmail.com (Y.-W.W.); poling@ntu.edu.tw (P.L.-K.); 3 Department of Electrical Engineering, National Taiwan University, Taipei 10617, Taiwan; 4 Department of Physical Medicine & Rehabilitation, National Taiwan University Hospital Hsin-Chu Branch, Hsinchu 302058, Taiwan

**Keywords:** entrapment neuropathy, animal model, hydrogel, ultrasound, carpal tunnel syndrome

## Abstract

Entrapment neuropathy (EN) is a prevalent and debilitative condition caused by a complex pathogenesis that involves a chronic compression–edema–ischemia cascade and perineural adhesion that results in excessive shear stress during motion. Despite decades of research, an easily accessible and surgery-free animal model mimicking the mixed etiology is currently lacking, thus limiting our understanding of the disease and the development of effective therapies. In this proof-of-concept study, we used ultrasound-guided perineural injection of a methoxy poly(ethylene glycol)-b-Poly(lactide-co-glycoilide) carboxylic acid (mPEG-PLGA-BOX) hydrogel near the rat’s sciatic nerve to induce EN, as confirmed sonographically, electrophysiologically, and histologically. The nerve that was injected with hydrogel appeared unevenly contoured and swollen proximally with slowed nerve conduction velocities across the injected segments, thus showing the compressive features of EN. Histology showed perineural cellular infiltration, deposition of irregular collagen fibers, and a possible early demyelination process, thus indicating the existence of adhesions. The novel method provides a surgery-free and cost-effective way to establish a small-animal model of EN that has mixed compression and adhesion features, thus facilitating the additional elucidation of the pathophysiology of EN and the search for promising treatments.

## 1. Introduction

Entrapment neuropathy (EN) is a prevalent and debilitative condition that causes pain, sensory impairment, and muscle atrophy in severe cases [1]. The etiology of EN is multifactorial and is hypothesized to be attributed to repetitive compression and shear stress injuries during motion [2,3,4]. Despite decades of research, the exact pathophysiology and the most effective treatment of EN are still debated. One of the reasons for this uncertainty is the lack of an easily accessible animal model that presents the complex, mixed pathological features of the disease.

Previously-used animal models can be classified into chemical and mechanical methods. In chemical methods, hypertonic dextrose or a proinflammatory agent is injected perineurally to induce inflammation and subsequent fibrosis [5,6]. Injection of hypertonic dextrose in the forepaws of rabbits has been used as a research model of carpal tunnel syndrome, the most common type of EN [5,6]. Being highly similar to the human carpal tunnel structurally, the rabbit model has potential for clinical translation. However, inflammation is not a key feature in EN, in which degeneration of myelin and perineural fibrosis mark the change [3,7]. Furthermore, multiple injections are required to induce obvious histology changes, partly attributed to brief injectate retention after injection. An injectate that can stay in situ after injection may be more suitable for the development of this model.

Mechanical methods include direct neural ligation with sutures [8], or perineural compression by angioplasty balloon or silicone cuff [9,10,11]. While offering consistently repeatable effects, these methods induce neuropathy predominantly owing to mechanical compression. The presence of balloon, cuff, or suture prevents direct interaction of the epineurium and surrounding connective tissue, a condition that is gaining attention as a significant contributing factor of EN [3,7]. Furthermore, the methods require surgery, which is time consuming and technically challenging. The tissue injury induced during surgical procedures can also complicate the pathogenesis.

Thermosensitive hydrogels, which are liquids in ambient temperature and convert to a gel state at physiological temperatures, could be injected in vivo and be retained in situ for up to several weeks [12]. We hypothesized that perineurally-injected hydrogel could encompass and compress the nerve by gelation, and that tissue reaction during resorption of the hydrogel could cause adhesion of the nerve and surrounding connective tissues, thus creating a mixed etiology model of EN. Therefore, the present study aimed to establish an easily accessible, surgery-free, and cost-effective small-animal model that presents a mixed etiology of EN by using ultrasound (US)-guided perineural injection of thermosensitive hydrogels, which form a gel state at physiological temperatures.

## 2. Results

### 2.1. US-Guided Perineural Injection

**Figure 1:**
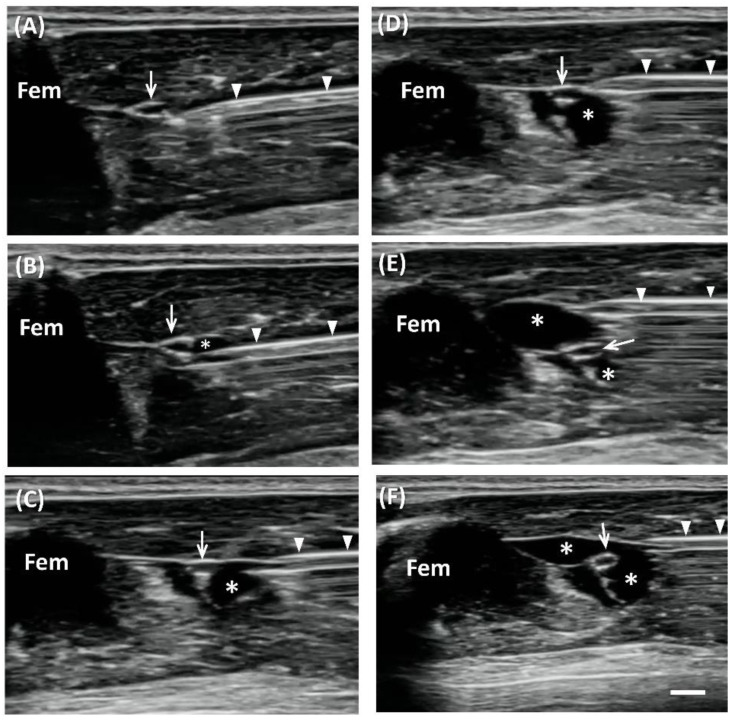
Ultrasound (US)-guided perineural injection of hydrogel. Short-axis view, in-plane approach of US-guided perineural injection of hydrogel (asterisks) showing the insertion of the needle (arrowheads) in the deep regions around the sciatic nerve (arrow) initially (**A**–**C**), and then superficially (**D**–**F**) (Fem, femur) (Scale bar: 1 mm).

**Figure 2:**
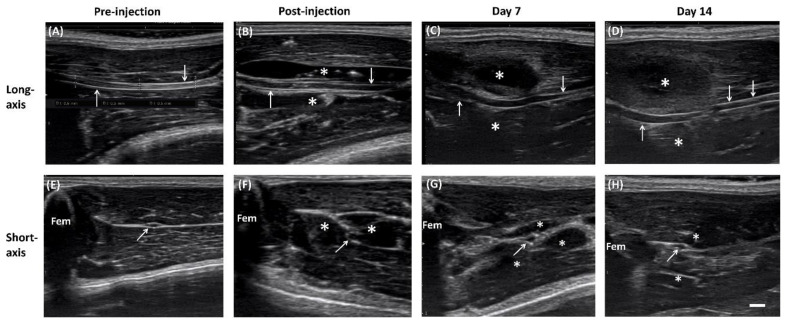
Sonography of sciatic nerve before and after injection. Long-axis (**A**–**D**) and short-axis (**E**–**H**) views of the sciatic nerve (arrows) before, immediately after, and 7 days and 14 days after hydrogel (asterisk) injection (Scale bar: 1 mm).

In total, 11 rats were used in the experiment. US imaging demonstrated perineural delivery of hydrogel, which appeared as homogeneous hypoechoic immediately postinjection, distributed both superficially and at deep sciatic nerve locations (Figure 1). The middle part of the sciatic nerve was completely surrounded by the hydrogel. Retention of perineural hydrogel at days 7 and 14 was confirmed sonographically. The hydrogel appeared to be slightly heterogeneous echogenic, possibly owing to the partial degradation and tissue reaction. Note that the surrounding hydrogel displaced the sciatic nerve, which became tortuous 7 and 14 days postinjection. In addition, the hydrogel-injected nerves appeared swollen proximal to the injection site, and thus demonstrated the pathognomonic feature of EN (Figure 2).

### 2.2. Changes of Sciatic Nerve Diameter

**Figure 3:**
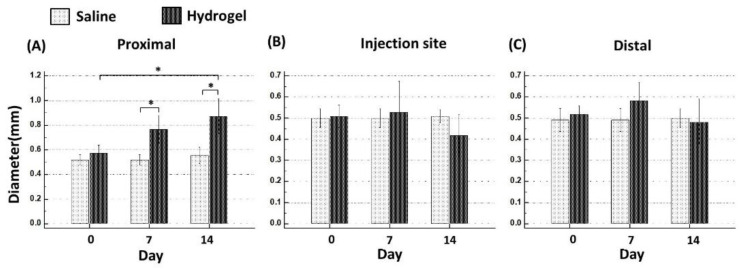
Changes of sciatic nerve diameter at the proximal (**A**), injection site (**B**), and at a distal (**C**) segment at days 7 and 14 (asterisk, *p* < 0.05).

The segment of the sciatic nerve proximal to the hydrogel injection site increased its diameter considerably at day 14 compared with the pre-injection status (*p* < 0.001). The diameter of the proximal segment was also larger than that of the saline group at days 7 and 14 (both *p* < 0.001). The diameter of the middle segment of the sciatic nerve (injection site) in the hydrogel group exhibited a tendency to become smaller at day 14 compared with that of the saline group (*p* = 0.06). The diameter of the distal segment of the hydrogel group remained unchanged when it was compared with the saline group or the pre-injection status (Figure 3).

### 2.3. NCV

Nerve conduction velocity (NCV) of the injected segment of the sciatic nerve was tested at day 14 after US evaluation. Considerable reduction of NCV by more than 50% was noted compared with that of the saline group (hydrogel group: 74.1± 12.3 m/s, saline group: 36.6 ± 7.8 m/s, *p* = 0.002). The results confirm that hydrogel injection resulted in impaired nerve conduction, a key feature of electrophysiological diagnosis of EN, in addition to morphological changes. Additionally, the encasement of the sciatic nerve by the hydrogel with the adhesion of the surrounding connective tissue was observed during the exposure of the nerve for the NCV procedure.

### 2.4. Histology

**Figure 4:**
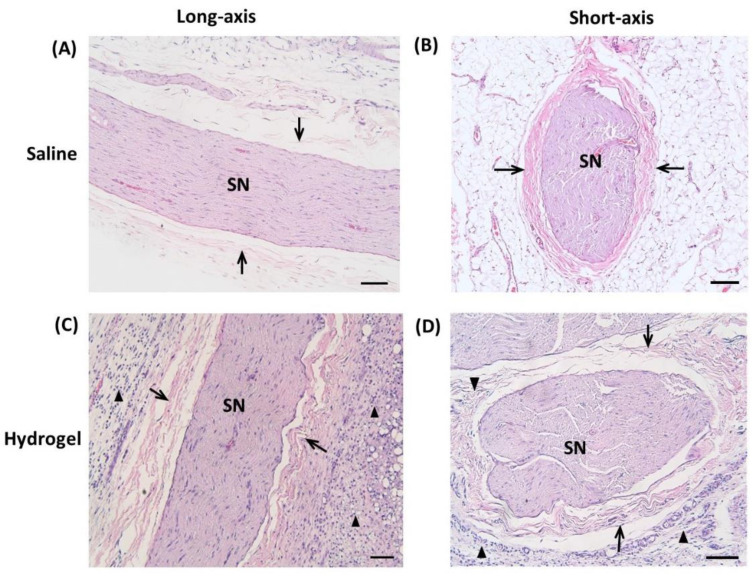
Representative micrographs of hematoxylin and eosin (H&E) stained sciatic nerve (SN) 14 days after injection of normal saline (**A**,**B**) and hydrogel (**C**,**D**). There is prominent cellular infiltration (arrowheads) surrounding epineurium (arrows) in the hydrogel-injected group with an irregular alignment of neural fascicles (scale bar: 100 μm).

**Figure 5:**
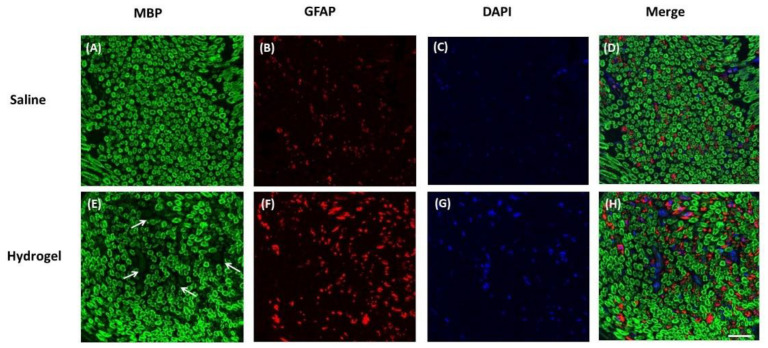
Representative micrographs of triple immunohistochemical staining of sciatic nerve, short-axis view 14 days after the injection of normal saline (**A**–**D**) and hydrogel (**E**–**H**). In the hydrogel group, the myelin basic protein (MBP) showed patchy areas (arrows) of breakdown (**E**), and glial fibrillary acidic protein (GFAP) showed increased expression (**F**). 4′,6-Diamidino-2-Phenylindole, Dihydrochloride (DAPI) was used for nucleic acid staining (scale bar: 40 μm).

Histologically, hydrogel-injected nerves had perineural infiltration of nuclear cells and deposition of irregular collagen fibers with disrupted fibrillary structure of axons at day 14. The saline-injected group showed well-organized axons and scarce nuclear cells perineurally (Figure 4). Triple immunohistochemical staining of sciatic nerves 14 days after hydrogel injection showed patchy breakdown of myelin basic protein (MBP) and increased expression of glial fibrillary acidic protein (GFAP) compared with those of the saline-injection group (Figure 5), indicating a possible demyelinating process.

## 3. Discussion

In this proof-of concept study, US-guided perineural injection of mPEG-PLGA-BOX hydrogel successfully induced sciatic nerve neuropathy, as confirmed sonographically, electrophysiologically, and histologically. The hydrogel-injected nerve appeared irregular and swollen proximally with slowed NCV across the injected segment, demonstrating the compressive features of EN. Histology demonstrated perineural cellular infiltration, deposition of irregular collagen fibers, and a possible demyelination process, indicative of the existence of adhesions. The novel method provides a surgical-free, minimally invasive way to establish small animal models of EN with mixed compression and adhesion features. The versatile model could be customized to create the EN of different mechanisms, representing different etiologies or stages of the disease, thus facilitating further understanding of the disease and the development of effective treatment.

The pathophysiology of EN is complex and multifactorial. Following chronic nerve compression, the nerves show disrupted and thinning of the myelin [2,13,14]. Decreased number and deformed collagen fibrils of perineural tissues was noted in patients with carpal tunnel syndrome [7]. Cross-sectional enlargement of the nerves and increased content of surrounding connective tissue is a common finding, even in subclinical individuals [15]. Of note, there is prominent thickening of the subsynovial connective tissue, without obvious inflammatory phenomenon [3,7,16], indicating a chronic degenerative feature of the disease.

While repetitive impingement of the nerve due to the increased interstitial pressure or external compression has been considered as the most important etiology, accumulating evidence has indicated the contributing role of shear stress caused by perineural adhesion and fibrosis [2,3,4,7,17]. Chronic impingement of the nerve results in perineural edema that impairs local circulation [2] that causes ischemia and reperfusion injury [18]. Surrounding connective tissue fibrosis and adhesion of the nerve ensue as a result of accumulated injury [3,7], which increase the shear stress of the nerve during motion and lead to additional damage [4].

In this regard, a model that presents both the compression and adhesion features of EN can describe the disease entity and may expedite further translational research. The proposed model presents a versatile choice of inducing EN with different characteristics, potentially by adjusting the composition of hydrogel or by combining different chemicals. For example, with quick gelation and long degradation times, the mPEG-PLGA-BOX hydrogel, as used in the present study, can induce compression-predominant EN by the mass effect (although tissue reaction may still cause perineural adhesion). By contrast, hydrogel with a lower degree of cross-linking has higher fluidity and longer gelation time, and tends to spread in a wider area rather than be retained focally after injection. By incorporating sclerosing or adhesive agents, adhesion-predominant EN could be simulated.

Sonographically, the injected hydrogel was retained perineurally on day 14. This is consistent with previous studies that reported the degradation rate of mPEG-PLGA-BOX. Peng et al. demonstrated that mPEG-PLGA-BOX was degraded by only 30% at 2 weeks in cultures in vitro [19]. In-vivo tests by Wu et al. showed that as a drug carrier, the hydrogel was able to provide steady drug release for at least 1 week, and the residual volume was still observable at day 14, and was measured to be approximately equal to 1/3 of the initial injection volume [20]. Longer degradation times were also possible by the modification of the degree of cross-linking of mPEG-PLGA. While the duration of the time needed to induce EN was highly variable, in most animal models there was an observable reduction of NCV and thinning of the myelin sheaths by week 2 based on histological evaluations [21]. Two in vitro studies showed that shear stress induced downregulation of myelin-associated glycoprotein and MBP, which are related to the demyelination process, and this occurred as early as a few hours after stimulation [22,23]. Our results indicate that the mPEG-PLGA-BOX hydrogel was retained long enough in vivo to produce morphological features of EN, which appeared as early as 7 days postinjection.

Recently, perineural injection of 5% dextrose (hydrodissection) has gained increased popularity as a treatment option in an attempt to release the adhesions between nerves and surrounding connective tissue [24,25,26,27]. However, controversy exists regarding the underlying mechanism and the optimal regiment of injection. As mentioned above, one of the reasons for the uncertainty is the lack of suitable animal models for testing the effects of injection therapy. Mechanical compression models by neural ligation [8], balloon, or silicone cuff [9,10,11] have generated tremendous insights into the pathophysiology of EN, but the presence of a balloon or cuff hinders the investigation of the perineural adhesion process and the injection effect. In this regard, our proposed method could serve as an optimal model to evaluate the efficacy of hydrodissection therapy. The hydrogel degraded gradually with controllable retention time. The increased perineural cellularity suggested the reaction of the tissue with the hydrogel, which resulted in possible adhesion. Based on the current results, our future study will optimize the hydrogel regimen with respect to the adhesion–induction properties and investigate the effect of hydrodissection on EN.

The study had several limitations. First, the time course of pathological changes of EN was not determined. Histology and NCV were evaluated at day 14 but were not sequentially examined. Needle stimulation electrodes had been evaluated in an attempt to measure NCV in vivo at day 7, but no consistent signals were obtained, possibly owing to the difficulty associated with the correct placement of the electrodes perineurally after hydrogel injection. Thus, NCV was only measured after the euthanasia of rats and dissection of the sciatic nerve at day 14. Second, the presence of compression and adhesions was demonstrated based on indirect morphological evidence, NCV, and histology. In future studies, the gliding resistance of the nerve could be measured before and after injection to demonstrate perineural adhesion. Furthermore, interstitial pressure could be measured to confirm the compression of the nerve owing to the hydrogel. Serial evaluation and longer follow-ups are also warranted to establish a comprehensive understanding of the disease process.

## 4. Materials and Methods

All experiments were performed in accordance with the guidelines established by the Institutional Animal Care and Use Committee at the National Taiwan University College of Medicine and the ARRIVE guidelines, and they were approved by the ethics committee of the Laboratory Animal Center at the National Taiwan University College of Medicine (Approval No. 20170470, 18 October 2018). All rats (Sprague–Dawley (SD), 6–8 weeks, weighing approximately 250–300 g) were procured from the National Laboratory Animal Center (Taipei City, Taiwan).

### 4.1. Experimental Protocols

US-guided hydrogel injection targeted the right sciatic nerves of male Sprague–Dawley rats at the mid-thigh level. The left sciatic nerves were injected with normal saline as the control group.

US imaging of bilateral sciatic nerves was conducted, and the diameter of the nerves was measured before injection and at 7 and 14 days after injection. The retention of the injected hydrogel was also confirmed by US scanning at day 7 and day 14.

**Figure 6:**
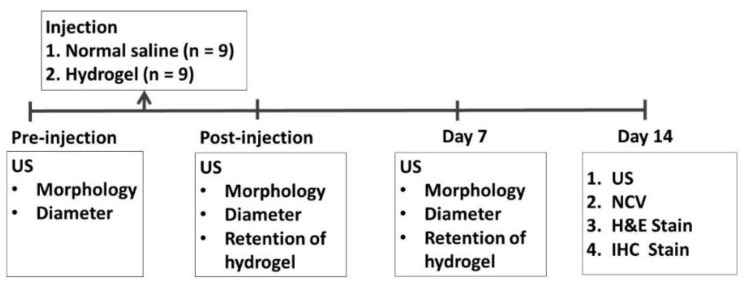
Schematic summary of the experimental procedure and outcome measures (US, ultrasound imaging; NCV, nerve conduction velocities; H&E, hematoxylin amd eosin; IHC, immunohistochemical).

The nerve conduction studies of the sciatic nerve were performed at day 14 after the rats were euthanized, by completely exposing the nerve proximal and distal to the injection site. The nerves were then harvested for histology (hematoxylin and eosin (H&E) staining) and immunohistochemical staining of the myelin basic protein (MBP), glial fibrillary acidic protein (GFAP), and nuclear staining with 4′,6-Diamidino-2-Phenylindole, Dihydrochloride (DAPI) (Figure 6).

### 4.2. US Evaluation and US-Guided Perineural Injection of Hydrogel

**Figure 7:**
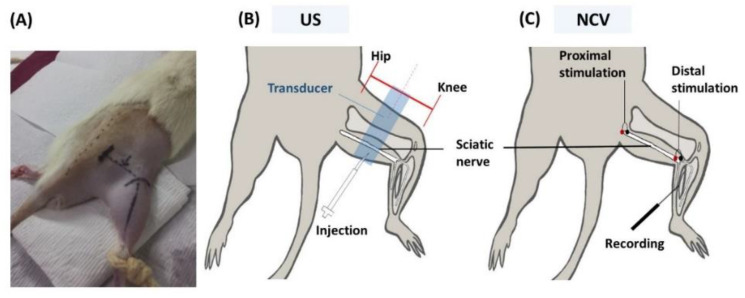
Positioning (**A**) and localization of sciatic nerve in US-guided injection (**B**) and NCV exam (**C**). Blue rectangle: placement of US probe.

US-guided perineural injection of 1 mL of hydrogel was performed by a physician who had 10 years of experience with US-guided intervention procedures. US-guided injection was performed with the use of a 15 MHz linear array transducer (L18-4, SONIMAGE^®^ HS1, Konica Minolta Healthcare Americas, Inc., Wayne, NJ, USA). The SD rat was anesthetized with isofluorane (2–3%, 2.1–3.3 L/min) and placed in a prone position with the hind legs fixed (Figure 7). The greater trochanter, knee joint, and the mid-point that connected these two sites were marked. Short-axis views were acquired first to locate the nerve, by placing the transducer perpendicular to the femur, with the center of the transducer aligned with the mid-point. The sciatic nerve appears as a hollow, small ovoid structure at the interfascial plane medial to the femur (Figure 1 and Figure 2).

The transducer was rotated 90° degrees to align with the femur to obtain the long-axis view of the sciatic nerve. The nerve appeared as a long, double contour structure. The mid-point of the transducer was aligned with the mid-point that connected the greater trochanter and knee joint. The diameter of the nerve was measured at proximal, middle, and distal sites.

US-guided injection was performed with short-axis acquisitions. The needle was inserted in plane with the transducer in a posterior–anterior direction and slowly advanced toward the nerve. Hydrogel or saline was injected after placing the needle tip between the nerve and fascia. The injectate (1 mL total) was dispersed in superficial and deep regions with respect to the nerve equally by repositioning the needle so that the injectate completely surrounded the nerve (Figure 1).

### 4.3. Preparation of mPEG-PLGA-BOX Hydrogel

The preparation of the mPEG-PLGA-BOX hydrogel has been described previously [19,28]. Briefly, the mPEG-PLGA copolymer was synthesized by mixing D, L-lactide (20 g) and glycolide (5.64 g) (both from Corbion PURAC Co., Provincie Zuid-Holland, Netherlands) with a mPEG (10.04 g) (Polysciences, Inc., Warrington, US) solution in a reactor. In total, 0.5 mL of tin (II) 2-ethylhexanoate or tin (II) octoate (Sn(Oct)_2_) solution (0.1 M concentration) was added to dried toluene under a nitrogen atmosphere at room temperature. The temperature of the reactor was then maintained at 160 °C for 9 h to generate the mPEG-PLGA copolymer.

The copolymer was dissolved in Dimethyl Sulfoxide (DMSO) followed by dialysis at 4 °C for 5 d, lyophilization for 2 d, and then it was mixed with 200 mL of DMSO in a three-neck reactor with a mechanical stirrer. The final volume of DMSO was adjusted to 90 mL. Succinic anhydride (SA) (1.84 g) was added under a nitrogen atmosphere to the mPEG-PLGA-DMSO solution at room temperature. The temperature of the reactor was maintained at 180 °C for 3 h. Finally, the linker 2,2′-Bis (2-oxazoline) (BOX) (1.28 g) (Tokyo Chemical Industry Co., Ltd. Toyko, Japan) was added to the reactor. The mPEG-PLGA-BOX copolymer solution was dissolved at 4 °C for 4 d, followed by lyophilization for 5 d. The rheological analysis and cytotoxicity were elaborated in our previous study [20].

### 4.4. Nerve Conduction Studies

The nerve conduction velocities (NCV) of the sciatic nerves were measured by dissecting the nerves to expose the segments of the injected sites, after the rats were euthanized with isofluorane (3.5–5%, 3.9–5.9 L/min). A pair of hooked-wire electrodes (micrograbbers #085-416800, Natus Neurology, Inc., Middleton, WI, USA) was placed distally and then proximally at the injection site to deliver electric stimulation. Needle electrodes (27 G, #019-476900, Natus Neurology, Inc., Middleton, WI, USA) were placed in the gastrocnemius muscle to record compound motor action potentials (CMAP) (Figure 2). An average of 10 CMAP was obtained for each stimulation site. Motor latency was measured as the mean duration from stimulation to the onset of CMAP. Thereafter, NCV was calculated by dividing the distance between proximal and distal stimulation sites by the difference of motor latency. All the recordings used electrodiagnostic equipment (Nicolet VIkingQuest, Natus Neurology, Inc., Middleton, WI, USA). 

### 4.5. Histology and Immunohistochemistry Staining

The rats were euthanized with isofluorane (3.5–5%, 3.9–5.9 L/min) at the end of the experiment, and the sciatic nerve was harvested and fixed with 10% formalin at room temperature overnight. The sample was embedded in paraffin and pasted on a glass slide (thickness = 7 μm), followed by the removal of paraffin, rehydration, and antigen retrieval (120 °C, 10 min). The sections were washed with tris buffered saline (TBS) that contained 0.025% Triton X-100 for 10 min and were blocked with 10% newborn calf serum and 1% bovine serum albumin in TBS for 2 h at room temperature. Primary antibodies were added to the sections to react overnight at 4 °C followed by the addition of fluorescence-labeled secondary antibodies. After 2 h at room temperature, the samples were washed with TBS and mounted with the EverBrite™ hardset mounting medium that contained DAPI to label the nuclei (1:5000, 40043, Biotium, Fremont, CA). The following primary antibodies were used: anti-MBP (1:1000, ab40390, Abcam, Cambridge, UK) and anti-GFAP (1:1000, Thermo Scientific, Waltham, MA, USA). The secondary antibodies used were Alexa Fluor 555-conjugated goat antimouse IgG (1:100; Thermo Scientific, Waltham, MA, USA) and Alexa Fluor 488-conjugated goat antirabbit IgG (1:100; Thermo Scientific, Waltham, MA, USA). Slides were viewed and images were captured with an LSM780 confocal microscope (Zeiss, Jena, Germany).

### 4.6. Statistical Analysis

All results are expressed as mean ± standard deviation. Normality was assessed using the Shapiro–Wilk test. The comparison between the hydrogel and saline groups was performed based on the Mann–Whitney U test for nonparametric nerve diameter data, and by an independent t-test for parametric NCV data. Wilcoxon two-sample tests were used to analyze the nerve diameter data collected at the follow-up compared with those at baseline. A test result was considered statistically significant at *p* < 0.05. All data obtained were analyzed with the MedCalc statistical software (version 15.11.3, MedCalc Software, Ostend, Belgium).

## 5. Conclusions

Ultrasound-guided perineural injection of hydrogel provides a surgery-free, minimally invasive method for the establishment of an animal model of EN of mixed etiology that could serve as a versatile tool for research on the pathophysiology and development of effective therapy of the disease.

## Data Availability

The datasets generated during and/or analyzed during the current study are available from the corresponding author upon reasonable request.
